# Behavior of *Vibrio* spp. in Table Olives

**DOI:** 10.3389/fmicb.2021.650754

**Published:** 2021-06-04

**Authors:** Guiomar Denisse Posada-Izquierdo, Antonio Valero, Francisco Noé Arroyo-López, Miriam González-Serrano, Alfonso M. Ramos-Benítez, Antonio Benítez-Cabello, Francisco Rodríguez-Gómez, Rufino Jimenez-Diaz, Rosa M. García-Gimeno

**Affiliations:** ^1^Department of Food Science and Technology, Agrifood Campus of International Excellence (ceiA3), University of Córdoba, Córdoba, Spain; ^2^Food Biotechnology Department, Instituto de la Grasa (IG-CSIC), University Campus Pablo de Olavide, Seville, Spain

**Keywords:** *Vibrio*, growth model, table olives, different NaCl concentrations, different pH levels, predictive microbiology

## Abstract

The presence of *Vibrio* species in table olive fermentations has been confirmed by molecular biology techniques in recent studies. However, there has been no report of any foodborne outbreak caused by *Vibrio* due to the consumption of table olives, and their role as well as the environmental conditions allowing their survival in table olives has not been elucidated so far. The aims of this work were to model the behavior of an inoculated *Vibrio* cocktail in diverse table olive environments and study the possible behavior of an inoculated *Vibrio* cocktail in table olives. First, an *in vitro* study has been performed where the microbial behavior of a *Vibrio* cocktail was evaluated in a laboratory medium and in olive brines using predictive models at different NaCl concentrations (2–12%) and pH levels (4.0–9.0). Afterward, a challenge testing was done in lye-treated olives inoculated at the beginning of fermentation with the *Vibrio* cocktail for 22 days. The *Vibrio* cocktail inoculated in table olives has not been detected in olive brines during fermentation at different pH levels. However, it was observed that this microorganism in a laboratory medium could reach an optimal growth at pH 9 and 2% salt, without time of constant absorbance (*t*_A_), and the maximum absorbance value (*y*_end_) observed was at pH 8 and 2% salt conditions. The statistical analysis demonstrated that the effect of salt concentration was higher than pH for the kinetic growth parameters (μ_max_, *t*_A_, and *y*_end_). On the other hand, it was confirmed that no growth of the *Vibrio* cocktail on any sample was noticed in lye-treated olive fermentations. Thus, it was concluded that the presence of olive compounds (unknown) did not allow the development of *Vibrio* strains, so it is a very safety product as it has a natural antimicrobial compound, but the possibility that a native *Vibrio* sp. is able to acquire the capacity to adapt to this compound should be considered in further studies.

## Introduction

Table olives are one of the major fermented vegetables in Mediterranean countries, with a worldwide production exceeding 2.5 million tons/year. The different types of elaboration are the result of the combination of tradition, artisan production, and technology, which go hand in hand to offer the best product to the consumer. Due to the great importance of this sector in the Mediterranean basin, it is essential to preserve conditions of quality and safety of this food to consumers (Perpetuini et al., 2020).

Olive is a fruit having a natural intense bitter flavor that is attributed to the content of the glycoside oleuropein. Therefore, the production process should remove this compound to improve table olives’ edibility prior to consumption. The Spanish-style traditional procedure for the elimination of bitterness consists of an alkaline treatment with NaOH, which produces the hydrolysis of oleuropein. Subsequently, a series of washings of the fruits are carried out to remove excess NaOH, and finally, they are immersed in a brine solution where they undergo lactic acid fermentation (Carmona et al., 2011).

*Vibrio* sp. is a very ubiquitous genus, which is widely distributed in fresh or salty aqueous environments, in coastal areas, and in estuaries. Currently, out of the existing 34 species, 12 are cataloged as human pathogens, and of these, 8 are transmitted by food (mainly of fish origin) and by contaminated waters (Austin and Austin, 2012; Joven, 2012). In recent studies, the use of molecular techniques allowed to identify the presence of *Vibrio* spp. in table olives during the fermentation process, and uncovering the role of this bacterium in table olives has gained more interest (Lucena-Padrós et al., 2014; Medina-Pradas and Arroyo-López, 2015; Benítez-Cabello et al., 2016). Some of the *Vibrio* species identified during the fermentation of table olives have been *Vibrio vulnificus*, *Vibrio furnissii*, and *Vibrio fluvialis* (Lucena-Padrós et al., 2015b; Benítez-Cabello et al., 2016). Recently, Lucena-Padrós et al. (2015a) have also reported the presence of a novel *Vibrio* spp. isolated from Spanish-style green table olives, named as *Vibrio olivae*, *V. furnissii*, and *V. fluvialis* give rise to acute gastroenteritis (Ramamurthy et al., 2014; Ballal et al., 2017), while *V. vulnificus* is related to systemic infections (Austin, 2010) and is associated with the consumption of raw oysters, being the leading cause of seafood-related deaths in the United States (Daniels, 2011). Despite the investigations that have been carried out, it is not clear yet as to the source of the presence of this bacterium in olive environments. Benítez-Cabello et al. (2019) performed a metataxonomic study on the bacterial diversity in table olive dressing, isolating *Vibrio* in the pepper dressing.

The species of the genus *Vibrio* are halophilic, so they require a minimum concentration between 1 and 3.5% of NaCl for growth, although the survival range is between 0 and 10%. Its optimal growth temperature is between 20 and 30°C, although some species can grow between 4 and 40°C (Kaysner and DePaola, 2004), and its optimal growth pH is between 7.8 and 8.6, although it can tolerate a wide range of pH levels covering between 4.8 and 11.0 (ACHIPIA, 2017).

To deepen our knowledge and understanding of microbial behavior against environmental factors and food processing conditions, predictive microbiology has emerged as a relevant tool for food safety management (Food and Agriculture Organization of the United Nations and World Health Organization [FAO and WHO], 2020). Predictive growth, survival, and inactivation models are based on the estimation of kinetic parameters [i.e., maximum growth and inactivation rates, lag time, maximum population density (MPD)] characterizing population dynamics as a function of environmental conditions. Most attempts at defining the growth of *Vibrio* species have considered temperature as a key factor either in broth (Miles et al., 1997) or in fish or seafood commodities (Parveen et al., 2008; Chen et al., 2011; Fernández-Piquer et al., 2011). However, environmental factors governing microbial behavior in table olive fermentation are mainly pH and salt, together with organic acids. Literature information about the predictive models of *Vibrio* against combinations of pH and NaCl levels is very limited, and most of the studies did not consider interstrain variability. To better understand the role of *Vibrio* species in table olives, development of mathematical models would be a very valuable instrument to implement food quality and safety measures. The objective of the present study was to evaluate the behavior of a cocktail of *Vibrio* spp. in the environment of table olives through the development of predictive microbiology models as a function of pH and NaCl conditions. Furthermore, the fate of *Vibrio* species in olive brines has been studied during table olive fermentation.

## Materials and Methods

### Experimental Design

The experimental study was conducted to determine the environmental conditions allowing growth or survival of *Vibrio* strains in table olives. The study was conducted consisting of two well-differentiated experiments: the first experiment was carried out in culture media (nutrient broth for *Vibrio* and olive brines) in Bioscreen C (Labsystems, Barcelona) so as to obtain growth curves at different parameter combinations, and the second experiment involved inoculation of pathogen in samples of table olives, which is intended to know how the behavior of *Vibrio* in table olives varies over time (22 days) and to study its relationship with lactic acid bacteria (LAB).

The first part of the study was carried out in a specific enrichment broth for the growth of *Vibrio* spp. at different pH levels (4, 5, 6, 7, 8, and 9) and NaCl concentrations (2, 4, 6, 8, 10, and 12%), making a full factorial design with a total of 36 conditions, with 10 replications each and all repeated three times. The microtiter wells were filled with 340 μl of modified culture medium. It should be taken into account that the enrichment broth used, alkaline saline peptone water (ASPW), contains 2% salt, so the condition with 2% salt is one in which NaCl has not been added to the medium. Microbial growth/survival was monitored through absorbance measurements in Bioscreen C, which measures bacterial growth over time by optical density (OD) measurements, for 7 days. Bioscreen C was programmed at a constant temperature of 30°C, with an hourly reading frequency and a wideband filter of 420–580 nm.

In addition, another set of experiment in Bioscreen was made at the same time in order to check if the real olive brine (extracted from sterilized jars with olives after 14 days in contact with the fruit and previously sterilized) was responsible for inhibiting growth. The real olive brine was characterized yielding the following levels: pH of 6.8, concentration of NaCl 6.28%, free acidity of 0.02, combined acidity of 0.05, and total sugars of 9.64% (sucrose 0.27%, glucose 6.26%, fructose 1.66%, and mannitol 1.45%). This real olive brine was modified in different conditions of pH levels (4, 5, 6, 7, 8, and 9) for the growth curve study.

The microtiter wells were filled with 340 μl of real olive and 10 μl of the *Vibrio* cocktail at a concentration of 10^5^ CFU/ml. Bioscreen C was programmed as described above.

At the second part of the experiment, the olives were inoculated with the pathogen and LAB. For this, four specific conditions have been proposed with their respective positive (samples inoculated with the target microorganism at time 0) and negative controls (non-inoculated samples):

• Olives inoculated with *Vibrio* spp. in non-sterile brine (V)
• Olives inoculated with *Vibrio* spp. in sterile brine (VS)
• Olives inoculated with *Vibrio* spp. and LAB in sterile brine (VBS)
• Olives inoculated with LAB in sterile brine (BS)

The non-sterile brine was used in one of the conditions to know the possible effect of the accompanying olive microflora on *Vibrio*. Each condition was performed three times.

### Preparation of the Microorganisms

Three strains of *Vibrio* spp., previously related to table olive fermentation, were used to carry out the study: specifically, *V. vulnificus* (CECT 529), *V. furnissii* (CECT 4203), and *V. fluvialis* (CECT 4217), which were obtained in lyophilized state from the Spanish Type Culture Collection of microorganisms (CECT) of the University of Valencia (Spain).

The lyophilized *Vibrio* spp. strains were resuscitated according to the manufacturers’ instructions and subsequently stored in commercial cryobeads at −20°C (Microbank^TM^, Pro-Lab Diagnostics, United States) until use. To reconstitute the strains, each cryobead was transferred to a test tube with 5 ml of brain-heart infusion broth (BHI, Oxoid, Basingstoke, United Kingdom), which was incubated for 24 h at 30°C. A second transfer was carried out in BHI, incubating at the same conditions, and finally, a third transfer culture was incubated for 16–18 h, thus allowing cells to reach late exponential state. The cocktail was made with equal parts of the three previously reconstituted strains of *Vibrio* spp. and, after typical morphology of *Vibrio* colonies, was then confirmed in selective agar media [CHROMagar^TM^ *Vibrio* (CHROMagar^TM^), *Vibrio* chromogenic agar (Condalab), and thiosulfate–citrate–bile salts–sucrose agar (TCBS agar, Oxoid)].

Subsequently, decimal dilutions were made taking 1 ml of the BHI broth with the grown strains and transferred to a test tube with 9 ml of saline (0.85%). To confirm the growth of the *Vibrio* cocktail, it was counted in the specific media using the spiral plater (Eddy Jet, IUL, Barcelona) and incubated at 30°C for 24 h.

### Predictive Models

Absorbance measurements obtained from the Bioscreen were processed in MS Excel. Once blanks were subtracted, average and standard deviation values were calculated for each tested condition.

The primary Baranyi model (Baranyi and Roberts, 1994) was fitted to the absorbance values using the DMFit Excel add-in. The estimated kinetic parameters corresponded to the time of constant absorbance (*t*_A_, h); maximum absorbance rate (μ_max_, OD/h), which corresponds to the increase of absorbance units per time unit; and the maximum absorbance value (*y*_end_, OD), which is the maximum absorbance level under the tested conditions.

Regarding *t*_A_, the calculation was based on a previously established absorbance detection limit (*D*_L_) of 0.2, i.e., the increase of absorbance in Bioscreen C in which growth was considered. We followed the approach of Vermeulen et al. (2007) where the detection limit corresponded to the sum of the absorbance of the blank plus three times the standard deviation of this value. Afterward, the *t*_A_ was obtained from each growth curve by calculating the difference between the absorbance value established as detection limit and the intercept with the *Y*-axis, then dividing the result by the slope of the curve.

To predict the behavior of *Vibrio* against pH and % NaCl conditions, polynomial secondary predictive models for *t*_A_, μ_max_, and *y*_end_ were developed in SPSS v25 (Chicago, Illinois, United States). Goodness of fit was assessed through the estimation of the coefficient of determination (*R*^2^) and root mean squared error (RMSE).

### Filtration Technique

To increase the limit of detection, a filtration technique was performed by passing the brine of the olive samples through a filtration ramp consisting of a funnel with a 0.22-μm membrane filter. The filter was placed on the TCBS selective medium on the opposite side through which the liquid passes. Finally, the plates were incubated at 30°C for 24 h and the colonies were counted.

Additionally, other filters were incubated in nutrient broth ASPW (Oxoid) to enhance the recovery of the damaged cells at 30°C for 24 h. Subsequently, this nutrient broth was cultured with the filters in TCBS medium and allowed to incubate at 30°C for 24 h.

### pH Measurements

For pH measurements, three non-inoculated samples were reserved for each condition and pH was periodically measured during the study period using a benchtop pH meter (HACH^®^, London, United Kingdom). In the case of the inoculated samples, the pH was measured with the Labbox (Madrid) test strips, with a wide measurement range of 1–14, to avoid contamination of the measurement equipment and, thus, cross-contamination of the samples.

### Olive Samples

The olive variety corresponded to Manzanilla-Cacereña, which was elaborated according to the Spanish style, which consisted in a lye treatment with NaOH (1.75%) for 7.30 h and washing with tap water for 4 h to remove excess alkali. Then, fruits were transported to the laboratory for packing in glass jars (60 g of fruits + 70 ml liquid) using a brine with 11% NaCl acidified with 0.2% HCl (30% purity). A total of 160 glass jars were obtained, which were then subjected to sterilization (or not according to experimental design) at 120°C for 15 min. Packed olives were kept under refrigeration (8°C) until their inoculation.

The samples of table olives in brine were obtained from the Instituto de la Grasa (IG-CSIC) located in Seville (Spain). Olives were subjected to a sterilization treatment and packed in glass containers with a capacity 125 ml. A total of 140 samples were made, where each glass jar contained 60 g of olive and 60 g of brine (11% NaCl), which was sterilized or not according to the case of the conditions studied: (V), (VS), and (VBS).

Upon arrival of the samples to the laboratory, pH was measured in order to check that the values were below 5.0 (to simulate the balance that normally occurs in the post-cooking product in an artisan elaboration). Therefore, the pH was adjusted and it was decided to add filter-sterilized NaOH to neutralize the pH to 7 since this pH would be too low to inoculate the *Vibrio*. During the study, the samples were stored at room temperature around 23°C.

### Inoculation of Olive Samples

Three strains of *Vibrio* spp., previously related to table olive fermentation, namely *V. vulnificus*, *V. furnissii*, and *V. fluvialis* (CECT 529, 4203, and 4217) were used, and three strains of lactic bacteria to simulate a bacterial group that initiates the fermentation of the product (LPG1, 13B4, and 119) are lactic bacteria of the homo-*Lactobacillus pentosus*.

Subsequently, decimal dilutions were made taking 1 ml of the BHI broth with the grown strains and transferred to a test tube with 9 ml of saline (0.85%). Samples of conditions V, VS, and VBS were inoculated with 0.1 ml of the *Vibrio* cocktail with a final concentration of 10^6^ CFU/ml. To confirm the growth of the *Vibrio* cocktail, it was counted in the specific media using the spiral plater (Eddy Jet, IUL, Barcelona) and incubated at 30°C for 24 h.

Lactic acid bacteria strains were commercially acquired (prototype patent pending) and reconstituted following the manufacturer’s recommendations. The necessary dilutions in phosphate buffered saline (PBS, Oxoid) were made to obtain a final concentration of LAB of 10^7^ CFU/ml in each sample. Sample inoculation was performed by adding 0.1 ml of the LAB solution to conditions VBS and BS.

### Microbiological Analyses

Microbial analyses were performed every 10–12 h during the first 3 days of fermentation and subsequently every 24–48 h until the end of the experiment. Each condition (V,VS,VBS, and BS) was analyzed in triplicate. Prior to analysis, the olive jars were shaken to homogenize the brine facilitating the resuspension of microorganisms. Subsequently, serial dilutions of the brine were made using 9 ml tubes of saline solution, and finally, the samples were surface plated using the spiral plater (Eddy Jet^®^ IUL, Barcelona, Spain). The Flash & Go (IUL) equipment was used for plate counting. The analyzed olive jars were discarded after analysis.

The study of the evolution of bacteria inoculated in the olive samples was carried out in the brine of the samples. For this, depending on the target bacteria of each condition of the sample (V,VS,VBS, and BS), the seeding was carried out in selective media. For *Vibrio* enumeration, TCBS *Vibrio*, CHROMagar, and *Vibrio* chromogenic agar were used at the incubation conditions of 30°C/24 h; for general counting, plate count agar (PCA, Oxoid) was used at 37°C/48 h; and for LAB, MRS was used at 33°C/48 h in anaerobiosis.

In order, to know the native microflora, mesophilic aerobic counts were performed on control samples by spiral seeding on PCA.

### Statistical Analysis

Data analysis was carried out using Microsoft Excel (Microsoft Corporation^®^) IBM SPSS v25 (United States) to carry out the estimation of the significant differences between the assayed variables:

– NaCl concentration and pH level in the culture medium
– Effect of pH in olive brine

To evaluate significant differences among independent variables, a non-parametric test, Kruskal–Wallis, was used; unfortunately, the data obtained were not relevant. A one-way ANOVA may yield inaccurate estimates of the *p* value when the data are very far from normally distributed. Like most non-parametric tests, it is performed on ranked data, so the measurement observations are converted to their ranks in the overall data set. Pearson’s correlation was also used; this coefficient is a measure of the strength of the association between two continuous variables. It is a type of correlation coefficient that represents the relationship between two variables that are measured on the same interval or ratio rank.

Significant differences were considered using a *p* value of 0.05.

## Results and Discussion

### Study of the Behavior of *Vibrio* spp. Under Different Conditions of pH and Salt Concentration in Bioscreen

**FIGURE 1:**
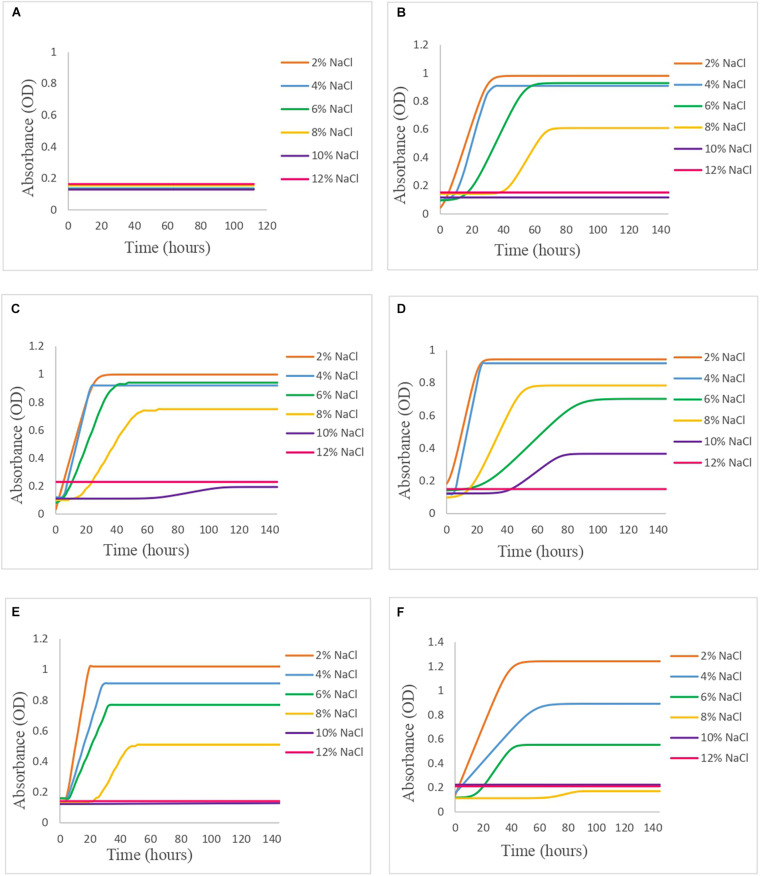
*Vibrio* spp. cocktail behavior in APWS broth modified with different % NaCl (2–12%) and pH (4–9) levels in Bioscreen: **(A)** pH 4, **(B)** pH 5, **(C)** pH 6, **(D)** pH 7, **(E)** pH 8, and **(F)** pH 9.

Once the OD data were obtained from Bioscreen, it was fitted to the primary growth models using DMFit. In Figure 1, it can be seen how the behavior in broth varies according to the concentration of NaCl and the pH level, and in Table 1, the growth parameters of each of the models are presented.

**TABLE 1:** Kinetic parameters of the growth models of *Vibrio* spp. in ASPW.

Growth conditions		μ_max_ (h^–1^)	t_A_ (h)	*y*_end_ (OD_420–580 nm_)	Primary model*	*R*^2^	SE (fit)

pH	NaCl%						
5	2	0.035	2.8	0.981	1	0.97	0.041
5	4	0.041	10.5	0.912	2	0.99	0.023
5	6	0.024	17.9	0.929	1	0.99	0.023
5	8	0.019	42.3	0.610	1	0.99	0.010
6	2	0.039	0.0	0.998	1	0.96	0.044
6	4	0.045	5.3	0.920	3	0.99	0.025
6	6	0.029	5.9	0.937	2	0.99	0.033
6	8	0.019	17.3	0.746	2	0.98	0.033
6	10	0.002	66.5	0.193	1	0.99	0.002
7	2	0.041	2.4	0.944	1	0.98	0.023
7	4	0.045	5.3	0.920	3	0.99	0.025
7	6	0.009	24.5	0.702	1	0.99	0.019
7	8	0.020	14.9	0.784	1	0.99	0.027
7	10	0.007	40.8	0.366	1	0.99	0.006
8	2	0.059	4.4	1.020	3	0.98	0.037
8	4	0.032	4.5	0.910	3	0.98	0.035
8	6	0.023	6.0	0.774	3	0.96	0.040
8	8	0.018	24.4	0.508	2	0.98	0.020
9	2	0.029	0.0	1.242	1	0.99	0.034
9	4	0.013	0.0	0.892	1	0.98	0.036
9	6	0.017	15.0	0.553	1	0.98	0.019
9	8	0.003	67.1	0.171	1	0.88	0.010

*1: DModel; 2: Baranyi and Roberts (scale-free); 3: Trilinear.

However, there were conditions in which the kinetic behavior was inactive or there was no growth, so they were not considered in the secondary model.

In the case of pH 4.0, there was no growth of *Vibrio* in any of the salt concentrations tested, and this was to be expected, since it is a pH too acidic for it to develop, since *Vibrio* is very sensitive to acidic pH (Wang and Gu, 2005; AESAN, 2010).

At pH 5.0, the highest concentrations of salt (10 and 12%) did not support the growth of *Vibrio*; however, at 2 and 4% of salt, a similar behavior of the slope of the exponential phase was observed (0.035 and 0.041 OD/h, respectively). In the case of 6 and 8% salt, the time of constant absorbance (*t*_A_) is delayed up to 18 and 42 h, respectively. As for the maximum absorbance value (*y*_end_), 2, 4, and 6% of salt present similar values, but at 8% of salt, the maximum absorbance value decreases significantly. At pH 6.0, the situation was similar to pH 5.0, but in this case, a slight growth of *Vibrio* was seen at the 10% salt concentration after 66 h of lag phase (Table 1).

As the condition gets closer to neutrality, pH 7.0, growth was observed at the concentration of 10% salt, after a lag time of 40 h, which was limiting to the rest of the pH values. In this case, it is seen that at the concentration of 8% salt, there is greater growth than at 6% salt, describing shorter lag time being the least although MPD was lower, which is the opposite of what occurs to the rest of the conditions (Table 1).

At pH 8.0, it was observed that the lag times of *Vibrio* at the salt concentrations of 2, 4, and 6% were similar (approximately 4.5–6 h); however, the MPD was different, with 1.020, 0.910, and 0.774 OD, respectively, and it was also observed that the slope of the exponential phase increases as the salt concentration decreases, describing the 2% salt condition as the best growth rate of all conditions with 0.059 OD/h (Table 1). In the case of 8% salt, the lag time was 24 h, with MPD well below the previous ones (0.508 OD). The concentrations of 10 and 12% salt at pH 8.0 limited the growth of *Vibrio*.

Regarding pH 9.0, growth has been observed in the conditions with 2, 4, and 6% salt, with the time of constant absorbance (*t*_A_) at the concentration of 2 and 4% salt being 0 h, while at 6% salt, it suffers a delay of 15 h (Table 1). In the case of 8% salt, a slight growth is described after 67 h of lag time, while at the concentrations of 10 and 12% salt, growth is inhibited. The best growth condition at this pH was by far 2% salt, with no lag time, with an exponential phase slope of 0.029 OD/h, and a very high MPD, being highest of all pH conditions (1.24 OD).

Comparing how the pH level affects the growth of *Vibrio* at the same concentration of salt (Figure 1), it can be observed that, at the condition of 2% salt and pH 9.0, the bacterium showed greater growth with a higher MPD than the rest under study, with its behavior at other pH levels being very similar, except at pH 4.0 where growth has not been described in any of the salt conditions tested. At 4% salt, the growth of *Vibrio* was affected at pH 9.0, as the slope in the exponential phase decreased as well as MPD. However, the remaining pH levels showed a similar behavior to the 2% salt condition. As the salt concentration increases (6 and 8% salt), this similar behavior at pH 5.0, 6.0, 7.0, and 8.0 is dispersing, until reaching the 10% salt concentration in which there is only significant growth of *Vibrio* at pH 7.0, after a time of constant absorbance (*t*_A_) of 40 h. At the 12% salt condition, there is no growth of *Vibrio* in any of the pH levels, so this condition was limiting.

In summary, according to our observations, it has been deduced that pH 4.0 is limiting for the growth of *Vibrio* in all studied salt conditions. The inhibition of the growth of *Vibrio* at pH 4.0 agrees with the study carried out by Mejlholm et al. (2012) in ready-to-eat shrimp brines, who found that the growth of *Vibrio* at pH values below 4.8 was inhibited.

The concentration of 12% salt is limiting for the growth of bacteria since growth is not described in any of the pH conditions. Twedt et al. (1969) described the behavior of *Vibrio* at different salt conditions (0–13%), with pH between 7.2 and 7.4, reporting a variable growth for the concentration of 10% of salt and an infrequent growth in the concentration of 13% of salt, describing growth in only 8 of the 79 samples studied, while in our study, there was no growth at the concentration of 12% of salt in any case, but at 10% salt, there was growth in the case of neutral pH.

At pH 5.0 and 6.0, though they did not correspond to the optimal growth pH range, *Vibrio* grew at most of the salt concentrations, apart from the highest ones (10 and 12% of salt). Whitaker et al. (2010) studied the behavior of *Vibrio* at different salt conditions (0.5, 1, and 3%) and pH levels (5.0 and 7.0) reporting that the growth of *Vibrio* at salt concentrations of 3% allowed a better adaptation to pH 5.0 compared with concentrations of 1% salt, and this may be an explanation for the fact of good growth at non-optimal pH levels for *Vibrio*.

### Modeling *Vibrio* Cocktail Growth

After obtaining the growth kinetic parameters in the primary models, a statistical analysis (ANOVA) of our variables was performed to see how they influence the kinetic parameters of the model. The results of this analysis revealed that pH level does not significantly affect (*p* > 0.05) any of the kinetic parameters (Table 2), while salt concentration does affect them highly (*p* ≤ 0.001) (Table 3).

**TABLE 2:** One-way ANOVA for pH in the kinetic parameters of *Vibrio* spp.

pH		Sum of squares	Degrees of freedom	Room mean square	F	*p* value
	Between groups	0.001	4	0	0.704	0.600
μ_max_	Within group	0.004	17	0		
	Total	0.005	21			

	Between groups	355.04	4	88.76	0.184	0.944
t_A_	Within group	8207.39	17	482.79		
	Total	8562.43	21			

	Between groups	0.052	4	0.013	0.145	0.963
*y*_end_	Within group	1.52	17	0.089		
	Total	1.57	21			

**TABLE 3:** One-way ANOVA for % NaCl on the kinetic parameters of *Vibrio* spp.

% NaCl		Sum of squares	Degrees of freedom	Room mean square	F	*p* value
	Between groups	0.003	4	0.001	7.859	0.001
μ_max_	Within group	0.002	17	0		
	Total	0.005	21			

	Between groups	5920.4	4	1480.1	9.523	0.000
t_A_	Within group	2642.1	17	155.4		
	Total	8562.4	21			

	Between groups	1.150	4	0.287	11.720	0.000
*y*_end_	Within group	0.417	17	0.025		
	Total	1.566	21			

On the other hand, a bivariate correlation was carried out, in which Pearson’s coefficient confirms that the concentration of salt is related to the three kinetic parameters of the model with *p* < 0.001, while pH has no correlation with any of them (Table 4). Miles et al. (1997) developed a growth model for *Vibrio parahaemolyticus* and showed that for the pH range of 6.5–8.9, growth rate was relatively constant, and therefore, pH did not influence growth.

**TABLE 4:** Correlations between the kinetic parameters of *Vibrio* spp. as a function of pH and% NaCl.

		μ_max_	t_A_	*y*_end_	pH	% NaCI
μ_max_	Pearson’s correlation	1	−0.705**	0.763**	−0.208	−0.791**
	Significance (bilateral)		0	0	0.353	0

t_A_	Pearson’s correlation	−0.0705**	1	−0.906**	−0.044	0.784**
	Significance (bilateral)	0		0	0.847	0

*y*_end_	Pearson’s correlation	0.763**	−0.906**	1	−0.119	−0.841**
	Significance (bilateral)	0	0		0.596	0

pH	Pearson’s correlation	−0.208	−0.044	−0.119	1	−0.059
	Significance (bilateral)	0.353	0.847	0.596		0.795

% NaCI	Pearson’s correlation	−0.791**	0.784**	−0 841**	−0.059	1
	Significance (bilateral)	0		0	0.795	

** statistically significant correlations at 95%.

To predict the behavior of the *Vibrio* cocktail as a function of pH and salt, a secondary predictive model was built, which will allow us to know the relationship between the kinetic parameters (maximum growth rate), time of constant absorbance (*t*_A_) and y_end_ (maximum absorbance value), and the ranges of pH and % NaCl studied. For the realization of this secondary growth model, those no-growth conditions were not included.

The mathematical Eq. 1 obtained for maximum growth rate (μ_max_) was as follows:

$$\mu_{\text{max}}=-0.049+\left(\mathrm{pH}\times 0.030\right)+\left(\%\mathrm{Salt}\times -0.001\right)$$

$$+\left(\mathrm{pH}\times \%\mathrm{Salt}\times -2\times 10^{-4}\right)+\left({\mathrm{pH}}^{2}\times -0.002\right)$$

(1) $$+\left(\%{\mathrm{Salt}}^{2}\times -2\times 10^{-4}\right)\left(R^{2}=0.76,\text{RMSE}=0.0085\right)$$

**FIGURE 2:**
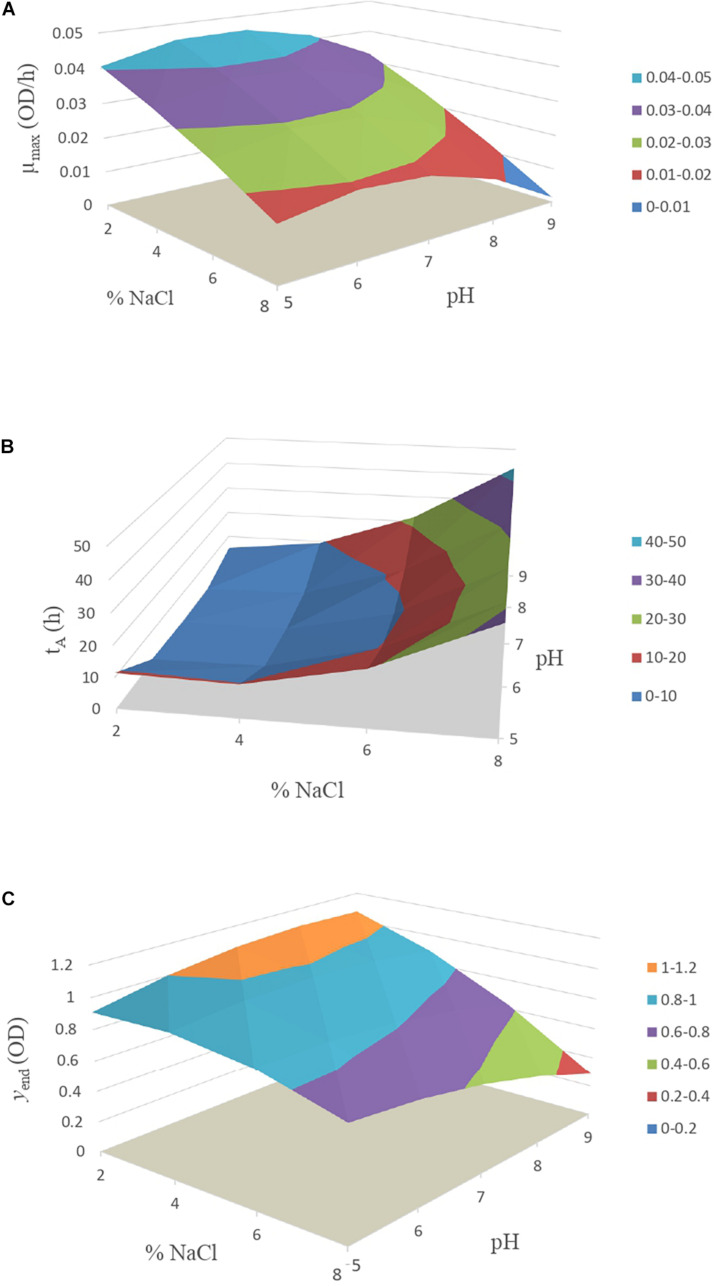
Response surface model of *Vibrio* spp. for kinetic parameter **(A)** μ_max_, **(B)** t_A_ and **(C)** y_end_.

In Figure 2A, the secondary model estimations of the μ_max_ can be seen, where the maximum growth rate decreased as salt concentration increases. Therefore, the greatest risk for the development of *Vibrio* in food would be in cases where high concentrations of salt are not reached, although in the case of table olives, this fact is unlikely due to its usual concentrations of 6–8% of salt.

Growth of *Vibrio* was not observed at any of the pH levels of the samples of real olive brine. The same occurred in the case of the study of LAB in the same condition. The concentration of salt of the real olive brine used as medium was 6.28%, which agrees with the values reported by Benítez-Cabello et al. (2016) at 14 days, due to the process of osmosis between brine and olive during processing.

This fact corroborates the result obtained in the first part of our study carried out on the samples of olives, where there was also no significant growth of any of these bacteria. All this makes us suppose that other antimicrobial substances that impede its development must be present, such as polyphenols present in olives, in which its antimicrobial action against various pathogenic bacteria has been described, including *Vibrio* (Isidori et al., 2005), since the physicochemical conditions would allow its multiplication according to our tests carried out in ASPW culture medium.

Regarding pH, the highest growth appears at the highest levels (7.0–9.0). Also, these pH levels are unlikely in table olives, because the level established for Spanish-style table olives by the IOC commercial standard is lower than 4.3. If lactic fermentation does not occur appropriately, pH levels below 5.0 would not be reached and *Vibrio* could develop according to the estimates of our model. Even so in the study carried out by Benítez-Cabello et al. (2016), viable *Vibrio* bacteria were identified during the entire fermentation process at pH < 4.3, and this could be attributed to the fact that they were adapted strains, so more studies would have to be done to verify *Vibrio*’s adaptation to acidic pH levels.

The mathematical Eq. 2 obtained for the time of constant absorbance (*t*_A_) was as follows:

$$t_{\text{A}}=168.22+\left(\mathrm{pH}\times -44.43\right)+\left(\%\mathrm{Salt}\times -9.76\right)$$

$$+\left(\mathrm{pH}\times \%\mathrm{Salt}\times 0.605\right)+\left({\mathrm{pH}}^{2}\times 2.976\right)+$$

(2) $$\left(\%{\mathrm{Salt}}^{2}\times 1.056\right)\left(R^{2}=0.76,\mathrm{RMSE}=0.4198\right)$$

In Figure 2B, the data obtained from Eq. 2 for the estimation of the time of constant absorbance (*t*_A_) of *Vibrio* are represented. The lower lag is described in those conditions where the salt concentration was less than 4% and pH was in the range of 5.5 to 8.0.

The mathematical equation (3) obtained for the maximum absorbance value (*y*_end_) was as follows:

$$y_{\text{end}}=-0.419+\left(\mathrm{pH}\times 0.334\right)+\left(\%\mathrm{Salt}\times 0.191\right)$$

$$+\left(\mathrm{pH}\times \%\mathrm{Salt}\times -0.024\right)+\left({\mathrm{pH}}^{2}\times -0.018\right)$$

(3) $$+\left(\%{\mathrm{Salt}}^{2}\times -0.01\right)\left(R^{2}=0.86,\mathrm{RMSE}=0.1180\right)$$

In Figure 2C, the maximum absorbance value (*y*_end_) is represented, obtained from Eq. 3. The situation which allowed the greatest multiplication of the bacteria was at 2% concentration of salt, starting from pH 6.0, under the studied conditions. On the other hand, the condition with the lowest growth was at 8% salt and pH level above 8.0.

A comprehensive bibliographic search has been carried out on the predictive models of *Vibrio* spp. existing to date. The results of these other studies are summarized in Table 5. None of these works were carried out on olives, but many of them were done in nutritious culture media or broths that simulated fishery products. The kinetic parameters in some cases were obtained by a rough estimate from the graphs provided in the scientific articles.

**TABLE 5:** Comparative summary of other predictive models of *Vibrio* spp.

	Model conditions				Kinetic parameters	
			
Models	pH	% NaCl	T (°C)	t_A_/Lag (hours)	μ_max_	*y*_end_
Hernández-Cabanyero et al. (2020)*	7	1	25	4	0.200 OD_62_5/h	0.76 OD_625_

Fujikawa et al. (2009)**	7	6.5	20	4	0.250 log N CFU/h	9.5 log N CFU
	7	8.9	20	18	0.300 log N CFU/h	9.3 log N CFU

Miles et al. (1997)*	7	9.6	20	ND	0.003 OD520	ND

Nishina et al. (2004)**	5.8	3	25	2	0.275 log CFU/h	8.5 log CFU/ml
	5.8	7	25	10	0.171 log CFU/h	8 log CFU/ml
	8	3	25	0	0.306 log CFU/h	9 log CFU/ml
	8	7	25	10	0.65 log CFU/h	8.5 log CFU/ml

Liu et al. (2017)*	8	0.5	37	ND	0.03–0.24 OD_(420–580 nm)_/h	ND
	8	3	37	ND	0.02–0.44 OD_(420–580 nm_/h	ND
	8	5	37	ND	0.01–0.26 OD_(420–580 nm)_/h	ND
	8	7	37	ND	0–0.15 OD_(420–580 nm)_/h	ND
	8	9	37	ND	0–0.12 OD_(420–580 nm)_/h	ND
	8	3	30	ND	0.005–0.065 OD_(420–580 nm)_/h	ND
	8	3	20	ND	0.007–0.031 OD_(420–580 nm)_/h	ND
	8	3	10	ND	0.001–0.014 OD_(420–580 nm)_/h	ND

ComBase (2019)**	6.72	3.5	20	0	0.105 log CFU/g	7.01 log CFU/g
	7.2	3.5	24.9	0	0.432 log CFU/g	7.01 log CFU/g
	7.2	4	21	0	0.148 log CFU/g	7.01 log CFU/g
	7.2	1	25.6	1	0.585 log CFU/g	9.34 log CFU/g

*ND: Undeterminated.*

The type of method used: *Optical Density (OD) measurements/**plate counts.

In general, the models generated by other authors contemplate pH levels that can be reached during the elaboration of table olives; however, this product requires a final pH of less than 4.3 for marketing. Regarding the concentration of salt, it can be observed that these models have been developed within a wide range ranging from 1 to 9.6%, presenting a variation in the kinetic parameter of the time of constant absorbance (*t*_A_) of 0–18 h, this period being of a high risk, since table olives are a low perishable product and do not present a significant deterioration of its organoleptic characteristics, causing a risk for the consumer. To the rest of the kinetic parameters, it was observed that *Vibrio* spp. present high growth rates and a maximum absorbance value if the conditions presented in Table 5 were found in table olives.

It is well known that measurement of microbial growth using turbidity is one of the most common methods for data collection and for estimation of microbial kinetic parameters, as shown in previous studies (Augustin et al., 1999; Nerbrink et al., 1999; Perni et al., 2005; Mytilinaios et al., 2012; Pla et al., 2015).

For *y*_end_, this parameter was set in OD units, and despite this limitation, *y*_end_ could give an approximation to the maximum population level at each condition tested, since we observed a clear decreasing trend at more limiting environmental pH and salt conditions.

Almost all models provided quite high goodness of fit with *R*^2^ 0.96–0.99 in 21/22 for growth curves and SE (fit) 0.002–0.044 for all growth curves, and these are similar to the values presented in ComBase of the Tasmanian Institute of Agriculture, University of Tasmania, Australia, for the kinetics of *V. parahaemolyticus*, strain: 38349. The samples were obtained using plate counts and modeling with the Baranyi and Roberts model, and this result provided goodness of fit *R*^2^ 0.997 and SE (fit) 0.108.

Various authors reported that measuring microbial growth by plate count and OD data gave similar results of kinetic parameters where high cell densities are used (Dalgaard and Koutsoumanis, 2001, 2013; Pla et al., 2015), such as in our case. Assuming the limitations of absorbance to build growth curves, it may be useful to help food microbiologists, researchers, and the food industry, so as to estimate the risk of *Vibrio* spp.

### Study of the Behavior of *Vibrio* spp. in Table Olive Fermentations

#### Evolution of pH

The evolution of pH throughout the fermentation of the inoculated and non-inoculated olives is shown in Table 6. In all conditions, the pH trend has been increasing, with fermentation starting at values of 6–6.5 and rising to 7.5–7.6 after 22 days of fermentation. This was due to the release of the NaOH previously absorbed by the olive pulp during lye treatment. In addition, these increased pH values may be attributed to the inhibition of LAB in the samples because of the high initial NaCl concentration present in the brines (11%) added at the beginning of the fermentation process. In this line, other authors (Durán et al., 1997) corroborated that NaCl concentrations higher than 8% in olive fermentations are practically inhibitory to LAB growth.

**TABLE 6:** Evolution of the pH measured by pH indicator test strips, under the different conditions studied in table olives.

	Inoculated samples				Non-inoculated samples			
		
Time (hours)	V	VS	VBS	BS	V	VS	VBS	BS
0	6	6	6.5	6.6	5.77	5.00	5.94	4.99
14	6.6	6.5	6.6	6.5	–	–	–	–
24	7	7	7	7	–	–	–	–
64	7.5	7.6	7	7	–	–	–	–
136	7	7	7	7	–	–	–	–
145	7	7.6	7.6	7.5	5.99	5.72	5.71	5.78
190	6.5	7.5	7	7	–	–	–	–
333	7.6	7.6	7.5	7.6	6.10	5.94	5.84	6.11
525	7.6	7.6	7.5	7.6	5.99	5.77	5.80	6.02

The results of the non-inoculated samples are lower than those obtained in the inoculated samples, but the statistical analysis revealed that the difference was not significant (*p* > 0.05). It can also be seen from Table 6 that the pH variation in the fermentation time was very small (*p* > 0.05).

The pH values obtained in the samples of this study are high for what normally occurs in the fermentation process of table olives, and the expected would have been a progressive decrease in pH due to the action of LAB due to the consumption of sugars and the production of lactic acid. The pH values of table olives after 20 days of fermentation are usually approx. 4.5 under normal conditions (Garrido Fernández et al., 1995). Although there is variability in the table olive production process, it is usually very artisanal and has wide differences depending on the type of the final product.

#### Evolution of Microbial Populations

After performing several analytical points of the brine of table olives, there was no growth of *Vibrio* spp. in any of the conditions, so it was decided to reinoculate the samples after 136 h of fermentation. In the following analyses, there was still no significant growth, so it was concluded that the conditions of the olive brine were adverse for *Vibrio* cocktail growth. Not satisfied with these results, we used the filtration technique to increase our detection capacity and also an enrichment stage to strengthen the growth of bacteria if they were injured or stressed, and thus, confirmation was obtained on the non-viability of bacteria.

The authors have not been able to find any study in the literature in this regard. Most studies on the behavior of *Vibrio* in food are focused on foods of marine origin, mainly seafood and fish. No studies on the behavior of this bacterium in olives have been found, so the results of this study reveal important information from an epidemiological point of view and demonstrate the originality of the work. In addition, in predictive microbiology databases, such as ComBase, where information is collected from growth models, more than 360 records can be observed, but none in these records focus on similar pH range and % NaCl although some are performed in growth medium as shown in Table 5.

The LAB strains have been isolated from the epidermis of table olive fermentations. The use of three strains makes the culture more robust and flexible and enables it to work in a greater number of conditions compared with other cultures formed by a single microorganism. These strains have a high capacity for adhesion to the surface of the fruit and can prevent colonization of the fruits by pathogenic or altering microorganisms through an exclusion mechanism. Despite this fact, LAB were not able to grow in the conditions of the olive samples, probably because the initial conditions of salt were not adequate for them (more than 6%).

## Conclusion

No growth of inoculated *Vibrio* cocktail was noticed in any of the samples analyzed with the presence of olive compounds. This makes us supposed that antimicrobial substances present in olives as polyphenols could prevent its development, even though there is always the possibility that autochthonous *Vibrio* strains could better adapt to these compounds. On the contrary, *Vibrio* growth was observed and modeled in a laboratory medium for diverse combinations of pH and salt that usually are present during olive fermentations, and it allows the estimation of kinetic parameters that can be useful for food industries to demonstrate the safety of their products. Finally, the statistical analyses revealed that pH level does not significantly affect (*p* > 0.05) any of the kinetic parameters, while the salt concentration does affect them in a significant way, with this factor being the most decisive for the growth of this bacterium. Further studies of the capacity of adaptation of salt of autochthonous *Vibrio* strains will be necessary to better evaluate its risk during table olive fermentations.

## Data Availability Statement

The original contributions presented in the study are included in the article/supplementary material, further inquiries can be directed to the corresponding author/s.

## Author Contributions

GP-I, RG-G, AB-C, FR-G, and FA-L: conceptualization. GP-I, RG-G, and AB-C: methodology. GP-I and AR-B: formal analysis. GP-I and MG-S: investigation. GP-I, FA-L, and RG-G: resources. GP-I and RG-G: original draft preparation. GP-I, RG-G, FA-L, and AV: writing—review and editing. AV and RG-G: supervision. FA-L, RG-G, and RJ-D: project administration and funding acquisition. All authors have contributed substantially to the work reported and have read and agreed to the published version of the manuscript.

## Conflict of Interest

The authors declare that the research was conducted in the absence of any commercial or financial relationships that could be construed as a potential conflict of interest.
